# Early changes in immune cell subsets with corticosteroids in patients with solid tumors: implications for COVID-19 management

**DOI:** 10.1136/jitc-2020-001019

**Published:** 2020-11-20

**Authors:** Jennifer L Marté, Nicole J Toney, Lisa Cordes, Jeffrey Schlom, Renee N Donahue, James L Gulley

**Affiliations:** 1 Genitourinary Malignancies Branch, Center for Cancer Research, National Cancer Institute, National Institutes of Health, Bethesda, Maryland, USA; 2 Laboratory of Tumor Immunology and Biology, Center for Cancer Research, National Cancer Institute, National Institutes of Health, Bethesda, Maryland, USA

**Keywords:** inflammation mediators, T-lymphocytes, immunomodulation, immunity, cellular

## Abstract

**Background:**

The risk–benefit calculation for corticosteroid administration in the management of COVID-19 is complex and urgently requires data to inform the decision. The neutrophil-to-lymphocyte ratio (NLR) is a marker of systemic inflammation associated with poor prognosis in both COVID-19 and cancer. Investigating NLR as an inflammatory marker and lymphocyte levels as a critical component of antiviral immunity may inform the dilemma of reducing toxic hyperinflammation while still maintaining effective antiviral responses.

**Methods:**

We performed a retrospective analysis of NLR, absolute neutrophil counts (ANCs) and absolute lymphocyte counts (ALCs) in patients with cancer enrolled in immunotherapy trials who received moderate-dose to high-dose corticosteroids. We compared paired presteroid and available poststeroid initiation values daily during week 1 and again on day 14 using the Wilcoxon signed-rank test. Associated immune subsets by flow cytometry were included where available.

**Results:**

Patients (n=48) with a variety of solid tumors received prednisone, methylprednisolone, or dexamethasone alone or in combination in doses ranging from 20 to 190 mg/24 hours (prednisone equivalent). The median NLR prior to steroid administration was elevated at 5.0 (range: 0.9–61.2). The corresponding median ANC was 5.1 K/µL (range: 2.03–22.31 K/µL) and ALC was 1.03 K/µL (0.15–2.57 K/µL). One day after steroid administration, there was a significant transient drop in median ALC to 0.54 K/µL (p=0.0243), driving an increase in NLR (median 10.8, p=0.0306). Relative lymphopenia persisted through day 14 but was no longer statistically significant. ANC increased steadily over time, becoming significant at day 4 (median: 7.31 K/µL, p=0.0171) and remaining significantly elevated through day 14. NLR was consistently elevated after steroid initiation, significantly at days 1, 7 (median: 8.2, p=0.0272), and 14 (median: 15.0, p=0.0018). Flow cytometry data from 11 patients showed significant decreases in activated CD4 cells and effector memory CD8 cells.

**Conclusions:**

The early drop in ALC with persistent lymphopenia as well as the prolonged ANC elevation seen in response to corticosteroid administration are similar to trends associated with increased mortality in several coronavirus studies to include the current SARS-CoV-2 pandemic. The affected subsets are essential for effective antiviral immunity. This may have implications for glucocorticoid therapy for COVID-19.

## Background

The neutrophil-to-lymphocyte ratio (NLR) is reflective of systemic inflammation and has been associated with poor clinical prognosis in a number of conditions, including malignancies and infections.^1–4^ Corticosteroids often play a role in managing the acute inflammatory aspects of these conditions by inhibiting both the synthesis and secretion of inflammatory cytokines and by their broad effects on immune cell trafficking and survival.^5–7^ At the time of this writing, COVID-19, the disease caused by SARS-CoV-2, has infected over 35 million people and caused over 1 million deaths worldwide.^8^ The question of corticosteroid use in COVID-19 is complex and has conflicting considerations for management of morbidities such as viral pneumonitis, acute respiratory distress syndrome (ARDS), and cytokine storm syndromes.^9^ Early interim guidance from the WHO recommended against the routine use of steroids.^10^ While steroid administration may mitigate lung inflammation, risks include suppression of antiviral immune response and decreased viral clearance. A review of studies of steroids in viral respiratory diseases showed increased mortality and secondary infections in influenza, decreased viral clearance in SARS and Middle East respiratory syndrome (MERS) coronavirus outbreaks, and the expected complications of avascular necrosis and diabetes.^11^ High doses of corticosteroids have been associated with prolonged viral shedding and decreased clearance in COVID-19, other coronavirus infections, and influenza.^12^ While these findings describe potential risks of steroids in the management of these infections, data on associated inflammation may indicate possible benefits. Liu *et al*^13^ have found NLR to be an independent prognostic indicator of disease severity in COVID-19. High NLR, a marker of systemic inflammation, was the most significant predictive factor for development of severe illness and may suggest a role for corticosteroids in disease management. A retrospective study of 150 confirmed COVID-19 cases in Wuhan, China, also found several predictors of mortality, such as increased ferritin and interleukin (IL)-6, which may be associated with virally induced hyperinflammation and may be amenable to steroid treatment.^14^ The recently published Randomised Evaluation of COVID-19 thERapY (RECOVERY) trial reported that 6 mg of dexamethasone per day reduced the 28-day mortality rate for patients hospitalized with COVID-19 by 17%.^15^ This survival benefit was primarily seen in patients requiring invasive mechanical ventilation, with a lesser degree of improvement for those on oxygen supplementation alone. There was no benefit seen in patients who did not require respiratory support, and the potential for detrimental effects in this subgroup was noted. There is an urgent need to investigate the risk–benefit balance as well as the optimal timing of corticosteroid use in COVID-19 to inform management guidelines. Particular areas of concern are the timing of steroid effects on immune cells and whether it is possible to reduce toxic hyperinflammation while still maintaining T cell-mediated antiviral immunity, which is critical to clearing virally infected cells. In this investigation, we analyzed changes in NLR and individual neutrophil and lymphocyte components in patients with solid tumors after starting corticosteroids. Studying these changes in this population may be particularly relevant because ‘important manifestations of severe COVID-19 infection are shared with neoplasia, namely inflammation, immune dysfunction, and coagulopathy’.^16^ In select patients with available data, we also evaluated associated changes in immune subsets by flow cytometry and serum cytokine levels.

## Methods

**Figure 1:**
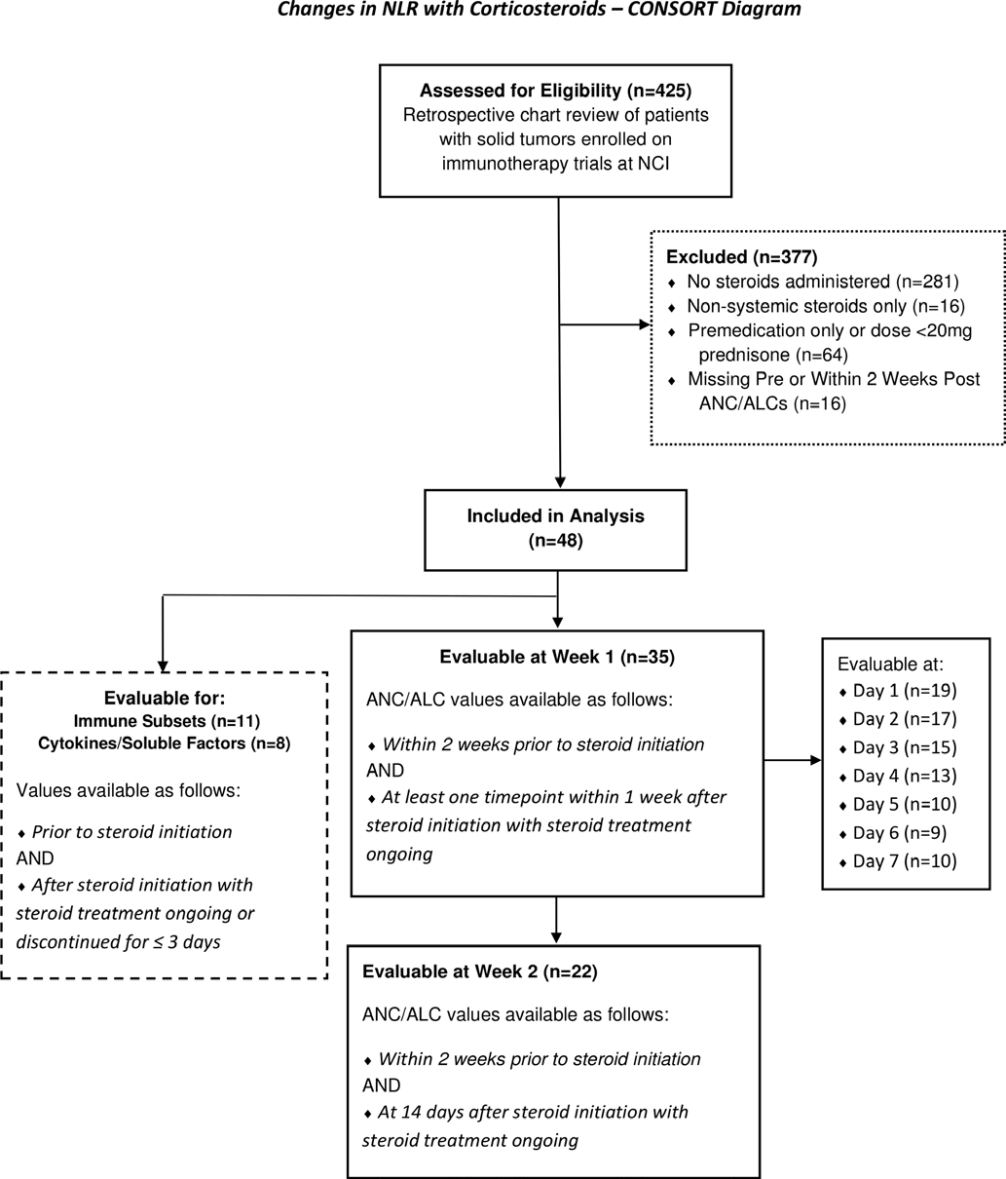
CONSORT diagram detailing the selection of evaluable subjects for analysis. ALC, absolute lymphocyte count; ANC, absolute neutrophil count; CONSORT, Consolidated Standards of Reporting Trials; NCI, National Cancer Institute; NLR, neutrophil-to-lymphocyte ratio.

We performed a retrospective analysis of clinical data obtained from patients (n=425) enrolled in 10 immunotherapy trials in solid tumors conducted at the National Cancer Institute (NCI) (online supplemental table 1). Patients were selected from this population for further analysis on the basis of confirmed moderate-dose to high-dose systemic corticosteroid administration, documented steroid start dates, and available data on absolute neutrophil count (ANC) and absolute lymphocyte count (ALC) prior to and within 14 days poststeroid initiation. Patients receiving only non-systemic steroids, premedication regimens, or low-dose courses were excluded (figure 1). For this analysis, a moderate to high dose of corticosteroids was defined as a starting dose of prednisone (or equivalent^17^) of ≥20 mg/day. ANC and ALC data were obtained from complete blood counts performed at the NCI’s Center for Cancer Research, and NLRs were subsequently calculated (datapoints with ALC=0 were excluded). Lab values were only included for analysis during active corticosteroid administration. Analyses of daily changes in NLR, ANC, and ALC for the first week after steroid initiation were performed in subjects with available data. Changes at 2 weeks after the start of steroids were also evaluated. Associated immune subset and cytokine data were investigated if available both prior to and after the start of steroid administration. Included post-timepoints were either collected during or immediately after (≤3 days) the conclusion of a steroid regimen. Multicolor flow cytometry was performed on frozen peripheral blood mononuclear cells (PBMCs) to identify up to 138 different immune cell subsets, including 10 classic subsets (CD4+ and CD8+ T cells, B cells, regulatory T cells (Tregs), natural killer (NK) cells, NK T cells, conventional dendritic cells (cDCs), plasmacytoid dendritic cells, monocytes, and myeloid-derived suppressor cells (MDSCs)), as well as up to 128 subsets relating to their maturation/function, as previously described^18–21^ (online supplemental table 2). Cytokine and soluble factor analysis was performed by ELISA on serum samples using commercially available kits according to the manufacturer’s instructions.

10.1136/jitc-2020-001019.supp1 Supplementary data

10.1136/jitc-2020-001019.supp2 Supplementary data

## Statistical analysis

For all parameters, paired presteroid initiation values were compared with poststeroid initiation values at each assessed timepoint using a Wilcoxon signed-rank test. These comparisons were performed for NLR and separately for the corresponding ANC and ALC values. P values of <0.05 were considered statistically significant. In both the peripheral immune cell subset and cytokine analyses, potentially biologically relevant changes were defined as those with a p<0.05 and a majority of patients with a >25% change. For the subsets, these parameters also included a difference in medians of presteroid initiation versus post >0.01% of PBMCs and a frequency >0.01% of PBMCs. All statistical analyses were performed using Stata SE V.16 or RStudio V.1.2.5001 software.^22 23^

## Results

We identified 48 patients who met the criteria of moderate-dose to high-dose corticosteroid administration with confirmed start dates and who had ANC and ALC data prior to and within 14 days poststeroid initiation. All patients had solid tumors and were enrolled in clinical trials of immunotherapy at the NCI; all but one had metastatic or locally advanced disease. Thirteen different tumor types were represented, nearly half (47.9%) accounted for by prostatic adenocarcinoma, cervical cancer, and other squamous cell malignancies. Median age was 60.1 years at the time of steroid initiation (range: 36.1–79.3); further demographics, including gender and race, are shown in table 1. Methylprednisolone, prednisone, or dexamethasone alone or in combination were administered to manage inflammatory toxicities (see table 1 for regimens and doses).

**Table 1:** Baseline characteristics

Baseline characteristics	n=48
Age (years): median (range)	60.1 (36.1–79.3)
Gender: # (%)	
Male	25 (52.1)
Female	23 (47.9)
Race/ethnicity: # (%)	
White	33 (68.8)
African-American	7 (14.6)
Asian	3 (6.3)
Hispanic	2 (4.2)
Other*	3 (6.3)
Malignancy stage: # (%)	
Metastatic/locally advanced	47 (97.9)
Non-metastatic	1 (2.1)
Corticosteroid regimen: # (%)	
Prednisone	29 (60.4)
Methylprednisolone and prednisone	15 (31.3)
Methylprednisolone	2 (4.2)
Dexamethasone and prednisone	1 (2.1)
Dexamethasone, methylprednisolone and prednisone	1 (2.1)
Dosing† (mg/24 hours): median (range)	
Prednisone	60 (20–160)
Methylprednisolone	60 (30–190)
Dexamethasone	12 (12–12)

*One each for unknown, multiple, American Indian/Alaska Native.

†Maximum dose at initiation of steroid tapers.

**Figure 2:**
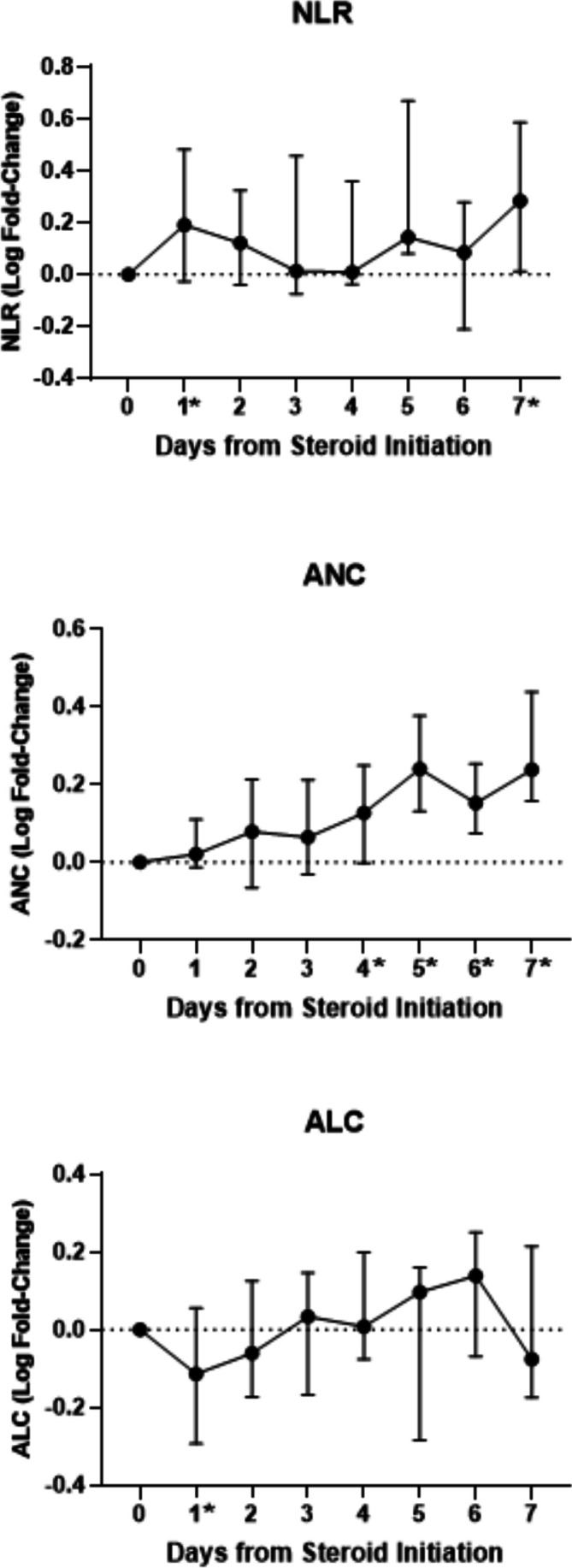
Median log fold change from presteroids to each post day 1–7 for NLR, ANC, and ALC is shown, along with the IQR. The medians and IQRs in this figure reflect all available values at each timepoint, as opposed to the paired pre and post values shown in table 2. Timepoints with significant changes from the paired analysis in table 2 are indicated here with an asterisk (*). ALC, absolute lymphocyte count; ANC, absolute neutrophil count; IQR, interquartile range; NLR, neutrophil-to-lymphocyte ratio.

At 5.0, the median NLR prior to steroid initiation was already elevated in these 48 patients (range: 0.9–61.2). The corresponding median ANC was 5.1 K/µL (range: 2.03–22.31 K/µL), and median ALC was 1.03 (0.15–2.57). Thirty-five patients had poststeroid values within the first week after treatment initiation; available matched pretreatment and posttreatment values were compared from day 1 to day 7 (table 2). Median NLR was increased at all week 1 poststeroid timepoints, but this increase was only significant by Wilcoxon signed-rank test on day 1 (10.8, p=0.0306) and day 7 (8.2, p=0.0273). ANC increased over the course of the first week, but this increase was minimal during the first 3 days and did not become statistically significant until day 4 (median 7.31, p=0.0171). Median ALC declined precipitously to 0.54 on day 1 poststeroids in the 19 patients evaluable at that timepoint (p=0.0243). This decline was transient and already recovering by day 2, though median lymphocyte counts remained below pretreatment levels through day 7. These ALC differences were not statistically significant outside of the drop on day 1. The median log fold change from presteroids to each poststeroid day 1–7 for NLR, ANC, and ALC is shown in figure 2, along with the interquartile range (IQR). Twenty-two patients had poststeroid values available at day 14, and both NLR (median 15.0, p=0.0018) and ANC (median 11.3, p=0.0000) were significantly increased relative to presteroid levels. Median ALC remained below pretreatment levels, but the decline was not significant (median 0.75, p=0.7024).

**Table 2:** NLR, ANC, and ALC during the first week after steroid initiation

Days from steroid initiation	N	Median	Mean	Range	SD	P value*
NLR (ANC/ALC)						
Pre	35	4.8	10.83	0.9–61.2	15.50	
1	19	10.8	13.1	1.1–47.2	11.40	**0.0306**
2	17	6	9.84	0.9–23.4	7.92	0.1522
3	15	8.3	14.02	0.5–61.9	15.98	0.1779
4	13	7.9	13.25	1.9–60.3	16.93	0.2656
5	10	7.1	42.41	2.5–190.2	74.29	0.0977
6	9	9.1	13.91	2.4–36.3	12.66	0.8203
7	9	8.2	19.19	4.9–94.6	28.71	**0.0273**
ANC (K/µL)						
Pre	35	5.05	6.50	2.03–22.31	4.40	
1	19	5.17	7.20	2.41–21.25	5.40	0.0874
2	17	6.23	7.13	1.71–16.86	3.67	0.1594
3	15	6.4	7.91	1.57–25.98	5.98	0.0833
4	13	7.31	8.01	3.67–27.72	6.25	**0.0171**
5	10	8.56	8.14	2.86–14.06	3.53	**0.0020**
6	9	7.76	9.97	3.34–26.39	7.18	**0.0039**
7	9	10.92	13.47	3.91–29.34	8.21	**0.0039**
ALC (K/µL)						
Pre	35	1.03	1.18	0.15–2.57	0.68	
1	19	0.54	0.84	0.22–2.29	0.67	**0.0243**
2	17	0.79	1.15	0.3–2.45	0.69	0.9265
3	15	0.77	0.99	0.35–2.86	0.70	1.0000
4	13	0.78	1.10	0.27–2.56	0.70	0.5417
5	10	0.83	1.01	0.06–3.12	0.89	0.3750
6	9	0.83	1.07	0.4–2.85	0.77	0.5703
7	9	0.95	1.26	0.31–3.63	1.00	0.8203

Thirty-five patients had labs drawn within the first week after steroid initiation. Available matched NLR, ANC, and ALC pre and post values were compared at each day after starting steroids through day 7. Numbers in bold indicate a p value <0.05

*Wilcoxon signed-rank test for paired pre and post values available at each timepoint.

ALC, absolute lymphocyte count; ANC, absolute neutrophil count; NLR, neutrophil-to-lymphocyte ratio.

Eleven of the evaluable patients had immune subset data available from their clinical immunotherapy trials that fulfilled the timepoint criteria. Subset data were collected at a median of 15 days (range: –41–0) prior to steroid initiation and 17 days (7–69) post. All patients remained on steroid tapers at the time of the postsubset data collection, except for two who had samples collected 1 and 3 days after completing steroids, respectively. Compared with prevalues, four classic subsets changed significantly with steroid administration: Tregs and cDCs decreased (p=0.0408 and 0.0020), while MDSCs and B cells increased (p=0.0029 and 0.0020). There was no significant change in overall CD4 and CD8 levels. However, 10 of the refined subsets showed significant changes, including decreases in CD4 T cells expressing the activation marker inducible T cell costimulator (ICOS) and CD8 effector memory T cells expressing the proliferative marker Ki67 (table 3).

**Table 3:** Significant changes in immune subsets by flow cytometry

Subset	Direction of change	Pre (median)	Post (median)	P value	>25% decrease (n)	>25% increase (n)	Total N
Treg	**↓**	1.08	0.34	0.0408	9	1	11
cDC	**↓**	0.25	0.09	0.0020	10	1	11
MDSC	**↑**	9.96	29.95	0.0029	0	9	11
B	**↑**	5.95	6.91	0.0020	0	9	11
CD4 CTLA4	**↓**	0.23	0.04	0.0067	8	1	11
CD4 ICOS	**↓**	4.33	1.26	0.0010	11	0	11
CD8 EM	**↓**	3.5	2.9	0.0322	8	1	11
CD8 EM ki67	**↓**	0.395	0.12	0.0431	5	0	6
Treg CD38	**↓**	0.275	0.045	0.0313	6	0	6
Treg CD49dneg	**↓**	0.43	0.16	0.0408	7	1	11
Treg ICOS	**↓**	0.51	0.15	0.0020	9	0	11
cDC Tim3	**↓**	0.14	0.04	0.0108	8	1	9
mMDSC	**↑**	9.23	17.15	0.0020	0	9	11
Non-classical monocyte	**↓**	1.275	0.36	0.0313	5	0	6

Eleven patients had immune subset data available presteroid and poststeroid initiation. Significant changes were defined by a p value <0.05, a median difference poststeroids versus presteroids >0.05% of PBMC and at least half of evaluated patients having a >25% change. The panels used for maturation/functional subset analyses were slightly different for the various immunotherapy trials, so certain subsets were not tested in all patients (n indicated for each).

cDC, conventional dendritic cells; EM, effector memory; ICOS, inducible T-cell co-stimulator; m, monocytic; MDSC, myeloid derived suppressor cells; Treg, regulatory T cells.

Eight of the evaluable patients had cytokine or soluble factor data available within the timepoint criteria, collected at a median of 22 days (range: –71–0) prior to steroid initiation and 15.5 days (9–69) post. Soluble CD40-ligand (sCD40L) significantly decreased with corticosteroid treatment (p=0.0312), with no other significant changes seen in other measures including soluble CD27 and IL-8. Only three of the subjects had Interferon (IFN)-γ data, and no significant changes were seen.

## Discussion

The risk–benefit calculation for corticosteroid administration in the management of COVID-19 is complex and urgently requires data to inform the decision, particularly in terms of the optimal timing of steroid initiation. It is critical to maintain a balance between suppressing toxic hyperinflammation and maintaining antiviral immune responses. Prolonged viral shedding and decreased clearance have been seen with high-dose corticosteroids in COVID-19,^12^ which may reflect impaired antiviral immunity. A study of 138 hospitalized COVID-19 patients in Wuhan, China, found that increasingly severe lymphopenia over time in the setting of increased ANC was associated with higher mortality.^24^ Glucocorticoids were administered to 44.9% of these patients. However, at the time of publication of that study, over half of the patients remained hospitalized, and no specific treatment-related outcomes were noted. While the RECOVERY trial showed a survival benefit with dexamethasone, that benefit was limited to patients requiring respiratory support.^25^ In this severely ill population, it is likely that any antiviral response had been overwhelmed, leaving toxic inflammation to dominate the clinical picture. This invites the question of optimal timing of steroid administration and the risk of suppressing antiviral immunity if given too early. Brun-Buisson *et al*^26^ found that corticosteroids increased the risk of death in ARDS associated with influenza A/H1N1 pneumonia by twofold to threefold when given within the first 3 days of the disease course, but not when given later. This effect remained significant after controlling for disease severity. A meta-analysis of 19 studies with 4916 patients with influenza also showed that corticosteroid treatment was associated with significantly increased mortality, particularly in the first 3 days after steroid initiation.^27^ This timing corresponds to the early and precipitous drop in ALC seen in this study. Investigations of other coronaviruses have also shown an association between lymphopenia and poor outcome. Virus-specific T cells were found to be necessary for clearance of infected cells in SARS-CoV, particularly in the lungs.^28^ A steady, gradual increase in lymphocytes over the course of approximately 2 weeks from symptom onset was seen in patients who recovered from MERS-CoV.^29^ It is clearly important to evaluate the early effects of corticosteroids not just on the NLR but also on the individual components of ANC and ALC as well as immune subsets.

The patients analyzed in this study already had an elevated NLR at baseline either as a consequence of inflammation from their disease, their immunotherapy, their steroid-requiring toxicity, or a combination of these factors. This helped to recapitulate the high NLR, hyperinflamed systemic environment of COVID-19 patients with moderate to severe illness. Saini *et al*^16^ also emphasized the similarities between malignancy and severe SARS-CoV-2 infection, particularly in terms of their cardinal features of inflammation, immune dysregulation, and coagulopathy. They went on to discuss the therapeutic implications of these shared characteristics, suggesting that the common pathophysiology provided a rationale for the use of anticancer drugs in the management of COVID-19, including corticosteroids. The shared underlying biology of these two complex conditions supports the extrapolation of the impact of steroids in this population of advanced cancer patients to those with moderate to severe COVID-19. The dexamethasone dose of 6 mg evaluated in the RECOVERY trial (prednisone equivalent of 40 mg) fell within the dose ranges examined in this analysis, and the results may inform the lack of improvement and potential for harm seen in patients who did not require respiratory support. Limitations of this study included a relatively small sample size and a heterogeneous population in terms of corticosteroid regimen and dosing. The complete blood counts, soluble factors and immune subset data may also have been confounded by administration of clinical trial immunotherapies, although these were typically on hold during steroid-requiring events. There was also potential for selection bias of more acutely or severely ill individuals, as steroids were not typically administered unless needed to mitigate symptoms of adverse events or disease progression. However, this selection may also have allowed the population to be more reflective of those hospitalized with SARS-CoV-2. The NLR timeline of interest was kept within 2 weeks poststeroid initiation due to the rapid deterioration of patients with COVID-19, with a median of 7 days from first symptom to hospitalization but only one additional day to ARDS.^24^ The most relevant changes were apparent very early. The statistically significant decrease in median ALC and the subsequent increase in NLR were seen 1 day poststeroid initiation. While the significant decrease in ALC proved to be transient, lymphocyte counts remained suppressed as much as 2 weeks after the start of steroid treatment. This is concerning given the importance of not merely maintaining but increasing lymphocyte counts to resolve COVID-19 infections. The timing of this sharp ALC drop also corresponds to the increased mortality seen with corticosteroids in early management (first 3 days) of influenza and ARDS.^26 27^ The continuing increases in ANC through week 1 and on to day 14 demonstrate a shifting of the contribution of neutrophilia to the NLR over time. The immune subset analysis further suggested potential impairment of antiviral responses through decreases in activated CD4+ cells expressing CTLA4 and ICOS. ICOS expression by CD4 cells is essential for optimal development of antibody responses to a number of viral infections, including influenza.^30^ Though the number of B cells increased, the CD4 cells necessary for their development of effective humoral responses declined. Chen *et al*^31^ specifically found this CD4 help to be required for antibody responses to COVID-19 infection. Steroid administration was also associated with significant decreases in cytotoxic effector memory CD8+ populations as well as the dendritic cells needed for antigen presentation. MDSCs significantly increased with corticosteroid treatment and are associated with decreased proliferation and IFN-γ production in both CD4+ and CD8+ T cells; monocytic MDSCs have been shown to suppress the B cell compartment as well.^32^ These findings are also concerning in the context of generating and maintaining antiviral immunity. The decrease in Tregs might initially be interpreted as beneficial by permitting more robust CD8+ T cell activity. However, Tregs may not play a major role in acute (as opposed to prolonged or chronic) viral infections, particularly those with high levels of inflammation such as COVID-19. The associated cytokines are inhibitory to Treg activation as well as differentiation from naïve precursors.^33^ The soluble factor data, while limited, did show a significant decrease in sCD40L, which is shed by activated T cells and platelets.^34^ This corresponds with the decreases in activated CD4+ T cells seen in the immune subset data. CD40-ligand plays a critical role in humoral immunity, with the decline after steroid initiation suggesting another impairment to development of effective antiviral antibody responses.^35^ Due to the limitations in the available cytokine data, reliable conclusions and correlations cannot be drawn at this time. However, the initial findings suggest that future evaluation of prospectively collected samples with expanded cytokine panels could provide valuable insights.

Several mechanisms may contribute to the early drop in ALC with persistent lymphopenia and the prolonged ANC elevation seen in response to corticosteroid treatment in this analysis. Supraphysiologic steroid administration is directly lymphotoxic through apoptosis mediated by the glucocorticoid receptor, while this process is inhibited in neutrophils.^36–38^ Corticosteroids also affect immune cell trafficking through direct effects on adhesion molecules and demargination, impaired migration to sites of inflammation, and redistribution of lymphocytes to the spleen, bone marrow, and lymph nodes.^39^ The phenomenon of demargination in lymphocytes may actually lead to an underestimate of the degree of lymphopenia and an overestimate of the number of lymphocytes in the tissue able to clear virally infected cells. However, naïve T cell populations seem to be more susceptible to killing by glucocorticoids than memory lymphocytes.^40^ Nevertheless, glucocorticoid administration leads to changes in ALC and ANC that are similar to those associated with increased mortality in several COVID-19 studies, including clinical characteristics seen in the current SARS-CoV-2 pandemic. Further characterization of the immune subsets suggests that the lymphopenia impacts CD4 and CD8 populations essential for antiviral immunity, and initial soluble factor results may be indicative of impaired antibody responses.

## Conclusions

These findings present factors to consider in the risk–benefit calculation of initiating corticosteroids early in the hospital management of COVID-19 before toxic inflammation has become overwhelming, as they may exacerbate already harmful trends in immune cell counts in these patients and specifically impair essential antiviral subsets and soluble factors. Alternate approaches targeting inflammatory cytokines or pathways (eg, siltuximab, an anti-IL6 monoclonal antibody, and anakinra, an IL-1 receptor antagonist) would allow virus-specific immune cells to remain and clear SARS-CoV-2 infected cells while still dampening the adverse clinical symptoms of a hyperinflammatory immune response.^41 42^ Timing is crucial, and steroids may be better reserved for situations where toxic inflammation has clearly overwhelmed any developing antiviral responses. If a decision is made to proceed with glucocorticoids, it should be weighed carefully against the need to clear virally infected cells, the patient’s current clinical condition, and other approaches that may not be as cytotoxic to lymphocytes.
