# Supplementary material for: Early changes in immune cell subsets with corticosteroids in patients with solid tumors: implications for COVID-19 management
Source: J Immunother Cancer. 2020 Nov 20;8(2):e001019. doi: 10.1136/jitc-2020-001019 (PMC7681794; doi:10.1136/jitc-2020-001019)
Supplement: Supplementary data [file jitc-2020-001019supp001.pdf]

Supplementary Table 1: Studies reviewed for patients meeting the analysis criteria.

**NCT #**	**n=425**
NCT01417546	69
NCT01772004	131
NCT02155647	6
NCT02517398	54
NCT02933255	16
NCT02840994	17
NCT02994953	27
NCT03384316	11
NCT03427411	56
NCT03493945	38

*Co-enrolled non-treatment protocols:
Biospecimen Acquisition (NCT00034216), Long Term Follow Up (NCT00451022)
