# Supplementary material for: Early changes in immune cell subsets with corticosteroids in patients with solid tumors: implications for COVID-19 management
Source: J Immunother Cancer. 2020 Nov 20;8(2):e001019. doi: 10.1136/jitc-2020-001019 (PMC7681794; doi:10.1136/jitc-2020-001019)
Supplement: Supplementary data [file jitc-2020-001019supp002.pdf]

Supplementary Table 2: 138 peripheral immune cell subsets analyzed by flow cytometry.

Ten parental phenotypes are identified as well as refined subsets of each relating to maturation and function. Expected function based on expression of specific markers within each subset is indicated in italics.

1. Total CD4$^+$ T cells

- PD-L1$^+$ CD4 - *activation/inhibition*
- PD-1$^+$ CD4 - *activation/inhibition*
- CTLA-4$^+$ CD4 - *inhibition*
- Tim-3$^+$ CD4 - *inhibition*
- 41bb$^+$ CD4 - *co-stimulation*
- Ki67$^+$ CD4 - *proliferation*
- ICOS$^+$ CD4 - *activation*
  - ICOS$^+$ PD-L1$^+$ CD4 - *activation/inhibition*
  - ICOS$^+$ PD-1$^+$ CD4 - *activation/inhibition*
- Total naïve (CCR7$^+$CD45RA$^+$) CD4
  - PD-L1$^+$ naïve CD4 - *activation/inhibition*
  - PD-1$^+$ naïve CD4 - *activation/inhibition*
  - CTLA-4$^+$ naïve CD4 - *inhibition*
  - Tim-3$^+$ naïve CD4 - *inhibition*
- Total central memory (CCR7$^+$CD45RA$^-$) CD4
  - PD-L1$^+$ CM CD4 - *activation/inhibition*
  - PD-1$^+$ CM CD4 - *activation/inhibition*
  - CTLA-4$^+$ CM CD4 - *inhibition*
  - Tim-3$^+$ CM CD4 - *inhibition*
  - Ki67$^+$ CM CD4 - *proliferation*
- Total effector memory (CCR7$^-$CD45RA$^-$) CD4
  - PD-L1$^+$ EM CD4 - *activation/inhibition*
  - PD-1$^+$ EM CD4 - *activation/inhibition*
  - CTLA-4$^+$ EM CD4 - *inhibition*
  - Tim-3$^+$ EM CD4 - *inhibition*
  - Ki67$^+$ EM CD4 - *proliferation*
- Total EMRA (CCR7$^-$CD45RA$^+$) CD4
  - PD-L1$^+$ EMRA CD4 - *activation/inhibition*
  - PD-1$^+$ EMRA CD4 - *activation/inhibition*
  - CTLA-4$^+$ EMRA CD4 - *inhibition*
  - Tim-3$^+$ EMRA CD4 - *inhibition*
  - Ki67$^+$ EMRA CD4 - *proliferation*

2. Total CD8$^+$ T cells

- PD-L1$^+$ CD8 - *activation/inhibition*
- PD-1$^+$ CD8 - *activation/inhibition*
- CTLA-4$^+$ CD8 - *inhibition*
- Tim-3$^+$ CD8 - *inhibition*
- 41bb$^+$ CD8 - *co-stimulation*
- Ki67$^+$ CD8 - *proliferation*
- Total naïve (CCR7$^+$CD45RA$^+$) CD8
  - PD-L1$^+$ naïve CD8 - *activation/inhibition*
  - PD-1$^+$ naïve CD8 - *activation/inhibition*
  - CTLA-4$^+$ naïve CD8 - *inhibition*
  - Tim-3$^+$ naïve CD8 - *inhibition*
- Total central memory (CCR7$^+$CD45RA$^-$) CD8
  - PD-L1$^+$ CM CD8 - *activation/inhibition*
  - PD-1$^+$ CM CD8 - *activation/inhibition*
  - CTLA-4$^+$ CM CD8 - *inhibition*
  - Tim-3$^+$ CM CD8 - *inhibition*
  - Ki67$^+$ CM CD8 - *proliferation*
- Total effector memory (CCR7$^-$CD45RA$^-$) CD8
  - PD-L1$^+$ EM CD8 - *activation/inhibition*
  - PD-1$^+$ EM CD8 - *activation/inhibition*
  - CTLA-4$^+$ EM CD8 - *inhibition*
  - Tim-3$^+$ EM CD8 - *inhibition*
  - Ki67$^+$ EM CD8 - *proliferation*
- Total EMRA (CCR7$^-$CD45RA$^+$) CD8
  - PD-L1$^+$ EMRA CD8 - *activation/inhibition*
  - PD-1$^+$ EMRA CD8 - *activation/inhibition*
  - CTLA-4$^+$ EMRA CD8 - *inhibition*
  - Tim-3$^+$ EMRA CD8 - *inhibition*
  - Ki67$^+$ EMRA CD8 - *proliferation*

3. Total Tregs

- PD-L1$^+$ Tregs - *activation/inhibition*
- PD-1$^+$ Tregs - *suppression*
- CTLA-4$^+$ Tregs - *suppression*
- ICOS$^+$ Tregs - *suppression*
- CD45RA$^+$ Tregs - *highly expandable in vitro*
- CD49d Tregs - *suppression*
- Ki67$^+$ Tregs - *proliferation*
- CD38$^+$ Tregs - *suppression*
- HLA-DR$^+$ Tregs - *suppression*

4. Total B cells

- PD-L1$^+$ B cells - *activation/inhibition*
- PD-1$^+$ B cells- *activation/inhibition*

5. Total NK

- PD-L1$^+$ NK - *inhibition*
- PD-1$^+$ NK - *activation/inhibition*
- Tim-3$^+$ NK - *activation/inhibition*
- Ki67$^+$ NK - *proliferation*
- NKp30$^+$ NK - *activation*
- NKp46$^+$ NK - *activation*
- NKG2D$^+$ NK - *activation*
- CD226$^+$ NK - *adhesion/activation*
- Total mature (CD16$^+$ CD56^{dim}) NK - *lytic*
  - PD-L1$^+$ mature NK - *inhibition*
  - PD-1$^+$ mature NK - *activation/inhibition*
  - Tim-3$^+$ mature NK - *activation/inhibition*
- Total functional intermediate (CD16$^+$ CD56^{br}) NK - *lytic, cytokine production*
  - PD-L1$^+$ functional intermediate NK - *inhibition*
  - PD-1$^+$ functional intermediate NK - *activation/inhibition*
  - Tim-3$^+$ functional intermediate NK - activation/*inhibition*
- Total immature (CD16$^-$ CD56^{br}) NK - *cytokine production*
  - PD-L1$^+$ immature NK - *inhibition*
  - PD-1$^+$ immature NK - *activation/inhibition*
  - Tim-3$^+$ immature NK - *activation/inhibition*
- Total unconventional (CD16$^-$ CD56^{dim}) NK - *non-lytic, non-cytokine production*
  - PD-L1$^+$ unconventional NK - *inhibition*
  - PD-1$^+$ unconventional NK - *activation/inhibition*
  - Tim-3$^+$ unconventional NK - *activation/inhibition*

6. Total NK-T

- PD-L1$^+$ NK-T - *inhibition*
- PD-L1$^+$ NK-T - *activation/inhibition*
- Tim-3$^+$ NK-T - *inhibition*
- Ki67$^+$ NK-T - *proliferation*

7. Total cDC

- PD-L1$^+$ cDC - *inhibition*
- PD-1$^+$ cDC - *activation/inhibition*
- Tim-3$^+$ cDC - *inhibition*
- Ki67$^+$ cDC - *proliferation*

8. Total pDC

- PD-L1$^+$ pDC - *inhibition*
- PD-1$^+$ pDC - *activation/inhibition*
- Tim-3$^+$ pDC - *inhibition*
- Ki67$^+$ pDC - *proliferation*

9. Total MDSC

- PD-L1$^+$ MDSC - *inhibition*
- PD-1$^+$ MDSC - *activation/inhibition*
- CD16$^+$ MDSC - *immature/suppression*
- Total monocytic (CD14$^+$ CD15$^-$) MDSC
  - PD-L1$^+$ mMDSC - *inhibition*
  - PD-1$^+$ mMDSC - *activation/inhibition*
  - CD16$^+$ mMDSC - *immature/suppression*
- Total granulocytic (CD14$^-$ CD15$^+$) MDSC
  - PD-L1$^+$ gMDSC - *inhibition*
  - PD-1$^+$ gMDSC - *activation/inhibition*
  - CD16$^+$ gMDSC - *immature/suppression*
- Total lineage negative (CD14$^-$ CD15$^-$) MDSC
  - PD-L1$^+$ lin neg MDSC - *inhibition*
  - PD-1$^+$ lin neg MDSC - *activation/inhibition*
  - CD16$^+$ lin neg MDSC - *immature/suppression*

10. Total Monocytes

- Classical monocytes - *phagocytic*
- Intermediate monocytes - *phagocytic/proinflammatory*
- Non-classical monocytes - *proinflammatory*
- PD-L1$^+$ monocytes - *inhibition*
- PD-1$^+$ monocytes - *activation/inhibition*

cDC, conventional dendritic cells; CM, central memory; CTLA-4, cytotoxic T lymphocyte-associated protein-4; EM, effector memory; EMRA, terminally differentiated effector memory; FoxP3, forkhead box P3; gMDSCs, granulocytic mononuclear derived suppressor cells; ICOS, inducible T cell co-stimulator; lin neg MDSCs, lineage negative MDSCs; mMDSCs, monocytic MDSCs; NK, natural killer; pDC, plasmacytoid DC; PD-1, programmed cell death-1; PD-L1, programmed cell death ligand-1; Tbet, T box expressed in T cells; TCR, T cell receptor; Tim-3, T cell immunoglobulin and mucin domain-3; Tregs, regulatory T cells.

Table adapted from reference [15], Donahue et al, Journal of Immunotherapy of Cancer, 2017, and reference [16], Lepone et al, Journal of Circulating Biomarkers, 2016.
